# Treatment of human hepatocellular carcinoma by the oncolytic herpes simplex virus G47delta

**DOI:** 10.1186/s12935-014-0083-y

**Published:** 2014-09-19

**Authors:** Jiani Wang, Lihua Xu, Weigen Zeng, Pan Hu, Musheng Zeng, Samuel D Rabkin, Renbin Liu

**Affiliations:** Breast Cancer Center, The Third Affiliated Hospital of Sun Yat-sen University, 600 Tianhe Road, 510630 Guangzhou, China; Department of Oncology and Hematology, The First Affiliated Hospital of Guangzhou Medical University, Guangzhou, China; Department of Colorectal Surgery, Cancer Hospital, Chinese Academy of Medical Sciences, Peking Union Medical College, 17 Panjiayuan Nanli, Chaoyang District, 100021 Beijing, China; State Key Laboratory of Oncology in South China, Sun Yat-Sen University Cancer Centre, Guangzhou, China; Department of Neurosurgery, Massachusetts General Hospital and Harvard Medical School, Boston, USA

**Keywords:** Hepatocellular carcinoma, Oncolytic herpes simplex virus, Cytotoxicity, Subcutaneous model

## Abstract

**Background:**

Oncolytic herpes simplex virus (HSV) can replicate in and kill cancer cells while sparing the adjacent normal tissue. Hepatocellular carcinoma (HCC) is amongst the most common and lethal cancers, especially in Third World countries. In this study, the cytotoxicity of a third-generation oncolytic HSV, G47Δ, was investigated in different human HCC cell lines and in an immortalized human hepatic cell line. Additionally, subcutaneous models of HCC were established to evaluate the *in vivo* anti-tumor efficacy of G47Δ.

**Methods:**

The HepG2, HepB, SMMC-7721, BEL-7404, and BEL-7405 human HCC cell lines and the HL-7702 human hepatic immortalized cell lines were infected with G47Δ at different multiplicities of infection (MOIs). The viability of infected cells was determined, and the G47Δ replication was identified by X-gal staining for LacZ expression. Two subcutaneous (s.c.) HCC tumor models of HCC were also established in Balb/c nude mice, which were intratumorally(i.t.) treated with either G47Δ or mock virus. Tumor volume and mouse survival times were documented.

**Results:**

More than 95% of the HepG2, Hep3B,and SMMC-7721 HCC cells were killed on by day 5 after infection with a MOI’s of 0.01. For the HL-7702 human hepatic immortalized cells, 100% of the cells were killed on by day 5 after infection with a MOI’s of 0.01. The BEL-7404 HCC cell line was less susceptible with about 70% cells were killed by day 5 after infection with a MOI’s of 0.01. Whereas the BEL-7405 HCC cells were the least susceptible, with only 30% of the cells were killed. Both the SMMC-7721 and BEL-7404 cells form aggressive sc tumor models. G47Δ replicates in the tumors, such that most of the tumors regressed after the G47Δ-treatment, and treated tumor-bearing mice survived much longer than the control animals.

**Conclusions:**

G47Δ effectively kills human HCC cells and an immortalized hepatic cell line at low MOI. Intra-tumor injection of G47Δ can induce a therapeutic effect and prolong the survival of treated mice bearing SMMC-7721 and BEL-7404 subcutaneously (s.c.) tumors. Thus, G47Δ may be useful as a novel therapeutic agent for HCC.

## Introduction

Hepatocellular carcinoma (HCC) is one of the most common and lethal malignancies worldwide [1–3]. It is more frequent among men than women, and the morbidity increases gradually with age. More than one million worldwide cases of HCC occur each year [4]. The incidence is highest in Third World countries, and a rising incidence has been recently been observed in developed countries.

The major etiological factors associated with HCC are infection with the hepatitis B (HBV) or C (HCV) viruses, chronic inflammatory liver lesions, necrosis of hepatocytes and subsequent fibrosis, long term exposure to high levels of AFBI or vinyl chloride in the diet, and heavy alcohol consumption [5]. At least one of these etiological factors can be identified, either alone or in combination with another factor [6–8]. Both HBV and HCV cause acute and chronic infections that are associated with over 80% of HCC cases worldwide, with most infected individuals remaining asymptomatic for many years [9,10]. It is estimated that approximately 10% to 40% of chronic HBV carriers eventually suffer from liver cancer [9,11].

Currently, several treatment modalities are considered to have curative potential: hepatic resection, liver transplantation, percutaneous radiofrequency ablation, and transarterial chemoembolization [12,13]. Recent progress in these treatment approaches has contributed to the improved survival rate of HCC patients [13]. Improvements in surgical techniques have led to significant advances in liver surgery, which have led to a substantial reduction in HCC mortality. The parallel development of laparoscopic surgery has also provided several advantages [14], although it is not routinely used [15].

However, there are limitations on these therapeutic options. HCC treatment depends on the stage of the tumor, the clinical characteristics, and the degree of liver dysfunction [16]. Patients must be diagnosed at an early stage and fulfill certain criteria. Unfortunately, HCC patients remain asymptomatic, and the majority of patients are not diagnosed until the tumor is in an advanced, non-resectable stage. Systemic chemotherapy is also disappointing, with low response rates and high toxicity. Treatment possibilities for these patients are limited. Hence, a new strategy that targets HCC specifically and effectively is needed.

One of the major goals of antitumor therapies is to target tumor cells selectively and specifically, while sparing adjacent healthy tissue from destruction. Oncolytic herpes viruses have been proposed as useful anti-tumor agents that kill dividing tumor cells but not normal tissue [17]. These viruses may be a new treatment strategy for malignant hepatic tumors.

Oncolytic herpes simplex viruses (HSVs) provide a potential therapeutic strategy that targets malignant tumors without damaging adjacent normal tissue. Replication of a single HSV can kill the host cell and release new virions, which can infect adjacent cells. Because these viruses destroy tumor cells by oncolysis, they have no cross-resistance with other therapy strategies, such as radiotherapy and chemotherapy; thus, they can serve as a complement to these therapeutic approaches.

Since oncolytic HSV was first described to treat brain tumors in 1991 [18], the unique biological characteristics of HSV have been improved by the development of additional HSV vectors that confer safety and specificity [19]. Over 20 different oncolytic HSV vectors have been evaluated in a large variety of different tumor types [19], including human pancreatic, gastric, and prostate cancer and mouse bladder and colorectal cancer [20–26]. Currently, six oncolytic HSV vectors, G207, G47∆, 1716, HF10, NV1020, and Oncovex^GM-CSF^ (talimogene laherparepvec) have progressed to clinical trials, with Oncovex^GM-CSF^ successfully reaching its primary endpoint in a randomized phase III trial for metastatic melanoma [19,27–29].

The virus investigated in this study is a third generation oncolytic HSV, G47Δ. It contains three principle mutations that result in its selective cytotoxicity to tumor cells. The γ34.5 gene precludes the shut-off of protein synthesis in host cells [19], and the γ34.5-deletion limits virus replication to cells lacking innate immune responses. The UL39 gene encodes for ICP6, the large subunit of viral ribonucleotide reductase, which is a key enzyme for deoxyribonucleic acid synthesis in nondividing cells [19]. A lacZ gene insertion in the UL39 gene inactivates it and blocks virus DNA replication in normal cells [21]. Deletion of the ICP47 gene places the US11 gene under the control of the immediate-early α47 promoter, which results in the amplification of γ34.5- mutant growth. Furthermore, the ICP47- mutation increases MHC class I presentation, which stimulates lymphocytes and decreases Natural Killer (NK) cytolysis of host cells [30]. These important features enhance the antitumor immune responses following treatment with G47Δ.

In this study, we investigated the cytotoxic effects of G47Δ on five human HCC cell lines and one immortalized human hepatic cell line. Furthermore, we evaluated the therapeutic effects of G47Δ in s.c. xenograft models of two human HCC cell lines.

## Materials and methods

### Ethics statement

The animal experiments in this study were conducted under the institutional guidelines of the Guangdong Province and approved by the Use Committee for Animal Care and the Sun Yat-sen University Institute Research Ethics Committee.

### Cells and virus

The HepG2 and Hep3B HCC cell lines (obtained from Dr. Qi Zhang, Laboratory of Hepatic Disease of the Third Affiliated Hospital of Sun Yat-sen University, Guangzhou, China) and Vero cells (African green monkey kidney, purchased from The Committee on Type Culture Collection of Chinese Academy of Sciences, Shanghai, China) were cultured at 37°C and 5% CO_2_ in DMEM with glucose (4.5 g/l, Mediatech, Inc., Herndon, VA) that was supplemented with 10% fetal calf serum (Hyclone Laboratories, Logan, UT). The SMMC-7721, BEL-7404, and BEL-7405 HCC and the HL-7702 human hepatic immortalized cell lines (obtained from Dr. Qi Zhang, Laboratory of hepatic disease of the Third Affiliated Hospital of Sun Yat-sen University, Guangzhou, China) were cultured at 37°C and 5% CO_2_ in RPMI1640 with glucose (4.5 g/l, Mediatech, Inc., Herndon, VA) that was supplemented with 10% fetal calf serum (Hyclone Laboratories, Logan, UT).

The G47Δ oncolytic herpes simplex virus was provided by MediGene, Inc. (San Diego, CA), and propagated in Vero cells cultured in DMEM containing 3% IFCS (inactivated fetal calf serum) at 34.5°C, as has been previously described. Briefly, the G47Δ viruses were diluted in PBS/1% IFCS. Vero cells were infected with a MOI of 0.02-0.03 and incubated in a 37°C (5% CO_2_) incubator. After 90 minutes, the viral inoculum was removed, DMEM/3% IFCS was added, and cells were incubated at 34.5°C (5% CO_2_) for approximately 2–3 days until total CPE occurred (the cells were rounded and refractive). Finally, the infected cells were harvested and resuspended in a 1:1 mix of DMEM (no serum) and the virus buffer [150 mM NaCl/20 mM Tris (pH 7.5)]. The cell suspension was rapidly frozen on EtOH/dry ice, and viral release was achieved by repeating the freeze-thaw process 3 times to lyse the cells. The cell debris was removed by low-speed centrifugation (2000 g for 10 min at 4°C). A plaque assay on the resultant G47Δ progeny was performed on Vero cells at 37°C to determine the viral titer.

### *In vitro* cytotoxicity

For cytotoxicity assay, cells were seeded in 6-well plates at 1 × 10^5^ cells per well. The cells were infected with G47Δ at a MOI of 0.01 and 0.1 when the cells were 50% confluent, while the controls were mock infected with PBS. After 24-hour incubation at 37°C, the number of surviving cells was counted everyday with a hemocytometer; the cells were washed twice with 1 ml of phosphate-buffered saline (PBS) to eliminate floating cells prior to counting. X-gal staining was performed daily, as described below, to show the infected cells.

### X-gal histochemistry

On days 1–5 post-infection, the culture medium was removed, and the cells were fixed with 0.2% glutaraldehyde/2% paraformaldehyde for 5 min. The cells were then washed 3 times with PBS and incubated with X-gal substrate solution [PBS (pH 7.2), containing 0.5 mg/ml 5-bromo-4-chloro-3-indolyl-β-D-galactopyranoside,5 mmol/L potassium ferricyanide, 5 mmol/L potassium ferrocyanide, 2 mmol/L magnesium chloride] at 37°C for 2 hours.

### Animal studies

Four-week-old male Balb/c nude mice were purchased from the Shanghai Institutes for Biological Sciences, CAS, Shanghai, China and bred with five mice in each cage. Each mouse was anesthetized with an i.p. injection of 0.60 to 0.80 ml 10% Chloral Hydrate. All the animal procedures were approved by the Sun Yat-sen University Institute Research Ethics Committee and the Use Committee for Animal Care. The mice were monitored daily for palpable (approximate 5-mm maximal diameter) tumor formation, and the tumors were measured using a Vernier caliper (the length was designated as “a”, and the width was designated as “b”). The animals were weighed twice weekly.

### Subcutaneous tumor treatment

The HCC SMMC-7721 (2 × 10^6^) and BEL-7404 (3 × 10^6^) cells were suspended in 100 μl of RPMI 1640 complete culture with 25% Matrigel (BD Biosciences) and implanted subcutaneously (s.c.) into the left flanks of 4-week-old nude mice. When the s.c. tumors were palpable, the s.c. tumors were inoculated twice weekly for two weeks with 2 × 10^7^/50 ul of G47Δ or with the virus buffer [150 mM NaCl, 20 mM Tris, (pH 7.5)] as a control. The tumor size was measured by Vernier calipers, and the tumor volume was calculated (V = a × b^2^/2). If the animals appeared moribund (lethargy, a hunched or recumbent posture, a rough coat or limited ambulatory movements in response to stimulation) or the maximal diameter of their tumors exceeded 18 mm, they were sacrificed and the date of death was recorded for the survival studies. The s.c. tumors were excised and then fixed in formaldehyde and embedded in paraffin for histological staining (hematoxylin-eosin).

### Virus biodistribution studies

The SMMC-7721 and BEL-7404 s.c. carcinoma-bearing Balb/c mice were treated 4 times with 2 × 10^7^ pfu/50 ul of G47Δ, as described above. The s.c. tumors were removed and embedded with tissue freezing medium and immediately frozen in dry ice. Cryostat sections (10 μm-thick) were prepared for biochemical staining of the LacZ enzyme. The sections were fixed with 2% paraformaldehyde in PBS for 10 minutes and washed with PBS 3 times for 15 min. Next, slides were incubated with PBS containing 2 mmol/L magnesium chloride, 0.01% sodium deoxycholate, and 0.02% NP40 for 10 minutes at 4°C. The sections were stained with a substrate solution [PBS (pH 7.2), containing 1 mg/mL 5-bromo-4-chloro-3-indolyl-h-D-galactopyranoside, 5 mmol/L potassium ferricyanide, 5 mmol/L potassium ferrocyanide, 2 mmol/L magnesium chloride, 0.01% sodium deoxycholate, and 0.02% NP40] at 34°C for 4 hours or over-night. The sections were washed with PBS/2 mmol/L EDTA and counterstained with eosin before mounting.

## Results

### *In vitro* cytotoxicity

**Figure 1:**
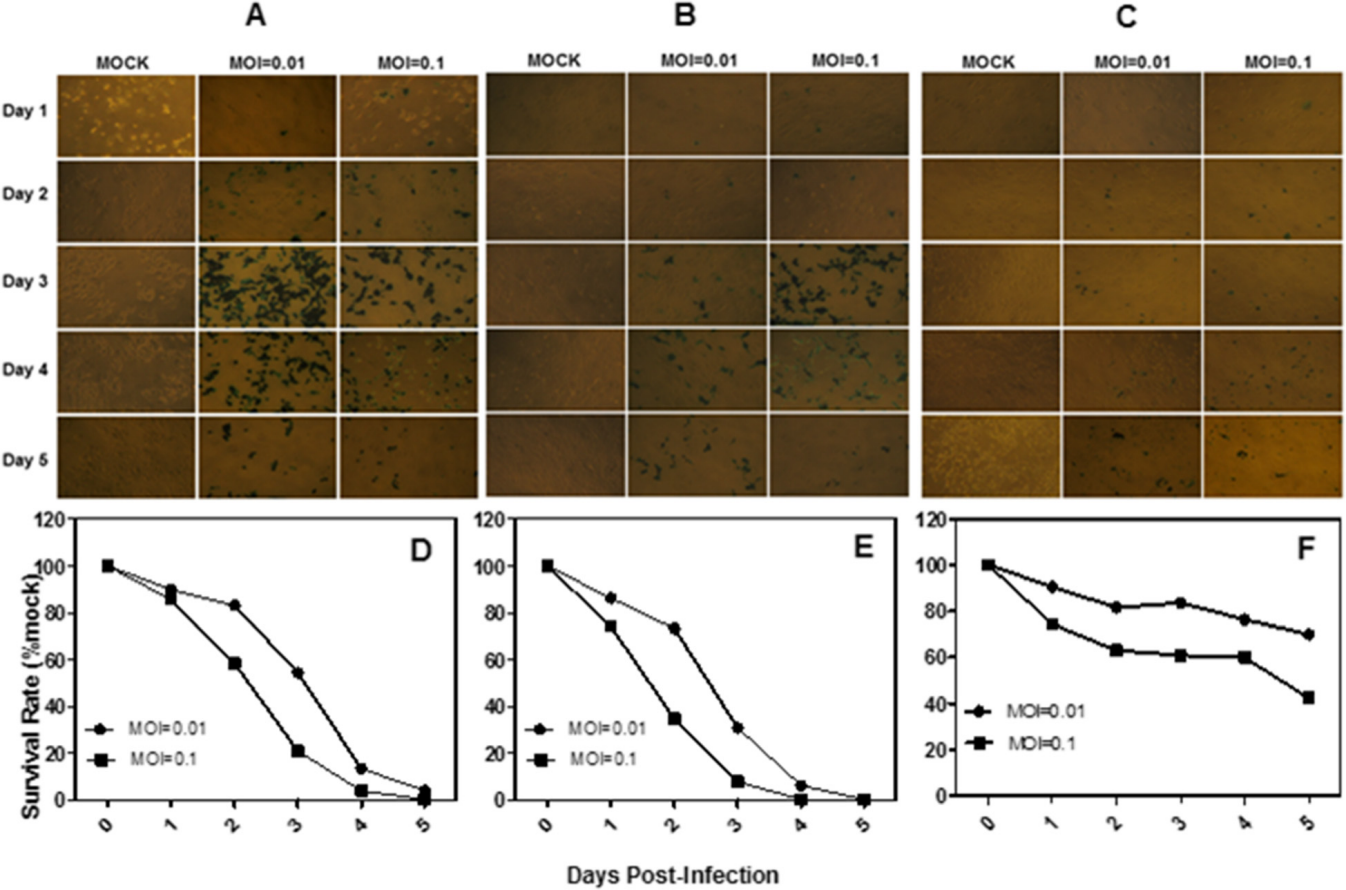
**The cytotoxicity of three HCC cell lines** ***in vitro*** **. A**, X-gal staining of HepG2 cells infected with G47Δ. Monolayers of HepG2 cells in 6-well dishes were infected with G47Δ or control vehicle and incubated with DMEM/1% heat-inactivated FBS at 37°C. On each of the 5 days following infection, the cells were stained with X-gal solution. The infected cells express Lac Z and stained blue. **B**, X-gal staining of Hep3B cells infected with G47Δ. **C**, X-gal staining of BEL-7405 cells infected with G47Δ. **D**, **E** and **F**, Monolayers of the HepG2, Hep3B and BEL-7405 cells in 6-well dishes were infected with G47Δ with MOI = 0.01 or MOI = 0.1, incubated in DMEM/1% heat-inactivated FBS at 37°C, and counted using a Coulter Counter on the days indicated. The average numbers of cells from duplicate wells are plotted as percentages of the mock wells.

**Figure 2:**
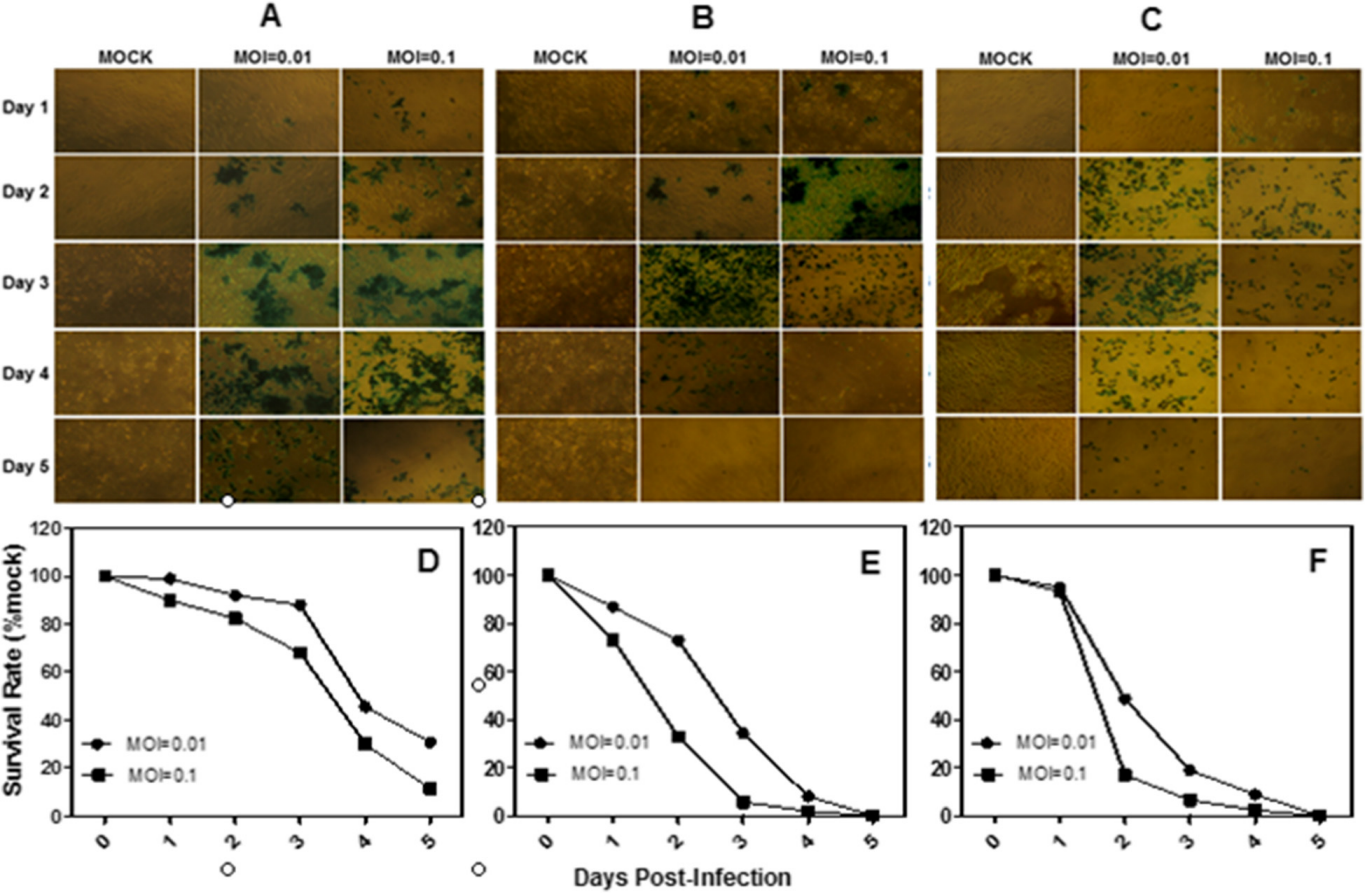
**The cytotoxicity of two HCC cell lines and one human hepatic immortalized cell line. A**, X-gal staining of BEL-7404 cells infected with G47Δ. Monolayers of BEL-7404 cells in 6-well dishes were infected with G47Δ or control vehicle and incubated with DMEM/1% heat-inactivated FBS at 37°C. On each of the 5 days following infection, the cells were stained with X-gal solution. The infected cells express Lac Z and stained blue. **B**, X-gal staining of SMMC-7721 cells infected with G47Δ. **C**, X-gal staining of HL-7702 cells infected with G47Δ. **D**, **E** and **F**, Monolayers of BEL-7404, SMMC-7721 and HL-7702 cells in 6-well dishes were infected with G47Δ with MOI = 0.01 or MOI = 0.1, incubated in DMEM/1% heat-inactivated FBS at 37°C, and counted using a Coulter Counter on the days indicated. The average numbers of cells from duplicate wells are plotted as percentages of the mock wells.

To assess the susceptibility of human HCC cells and a hepatic immortalized cell line to oncolytic HSV G47Δ cytotoxicity, monolayers of the HepG2, Hep3B, SMMC-7721, BEL-7404, and BEL-7405 human HCC cells and the HL-7702 human hepatic immortalized cell line were infected with G47Δ at low MOI’s (MOI = 0.01, MOI = 0.1). By day 5, more than 95% and 100% of the HepG2, Hep3B and SMMC-7721 HCC cells had been killed after infection with MOI’s of 0.01 and 0.1, respectively (Figure 1D, E and Figure 2E), similar to the HL-7702 human hepatic immortalized cells (Figure 2F). A slight decrease in effect was observed in the BEL-7404 hepatocarcinoma cells; more than 70% and 90% of the cells had been killed by day 5 after infection with MOI’s of 0.01 and 0.1, respectively (Figure 2D), while the BEL-7405 hepatocarcinoma cells were less sensitive, 29.9% and 57.7% of the cells killed by day 5 after infection with MOI’s of 0.01 and 0.1, respectively (Figure 1F). The infected cells were stained by X-gal histochemistry (G47Δ contain the lacZ transgene in the ICP6 region). The infected cells and the spread of the G47Δ viruses are shown by the blue staining in Figure 1A, B and Figure 2A, B.

### Efficacy of G47Δ on s.c. hepatocarcinoma tumors

**Figure 3:**
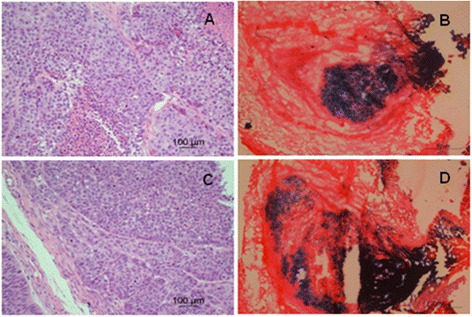
**Established s.c. tumors from HCC SMMC-7721 and BEL-7404 cells.** The cells were suspended in 100 μl of RPMI 1640 complete culture with 25% Matrigel (BD Biosciences) and subcutaneously implanted into the left flanks of 4-week-old nude mice. When the s.c. tumors were palpable, mice were inoculated into the s.c. tumor twice a week with 2 × 10^7^/50 ul of G47Δ or virus buffer [150 mM NaCl, 20 mM Tris, (pH 7.5)] for control. H-E staining of SMMC-7721 **(A)** and BEL-7404 **(C)** s.c. tumors (×200). Coronal sections through SMMC-7721 **(B)** and BEL-7404 **(D)** s.c. tumors 3 days after G47Δ (2 × 10^7^ pfu) injection to illustrate the virus replication in the s.c. tumors. Sections were stained with X-gal, and counterstained with eosin to identify cells containing replicating G47Δ (blue).

The SMMC-7721 and BEL-7404 cells were used for s.c. implantation *in vivo.*. S.c. tumors were established in the left flank of male Balb/c nude mice, followed by intratumoral injection with G47Δ when the tumors were palpable. To evaluate virus replication *in vivo,* the G47Δ-treated mice were sacrificed, and X-gal histochemistry was performed on the sectioned s.c. tumors. The blue-stained areas represent G47Δ replication (Figure 3B, D).

**Figure 4:**
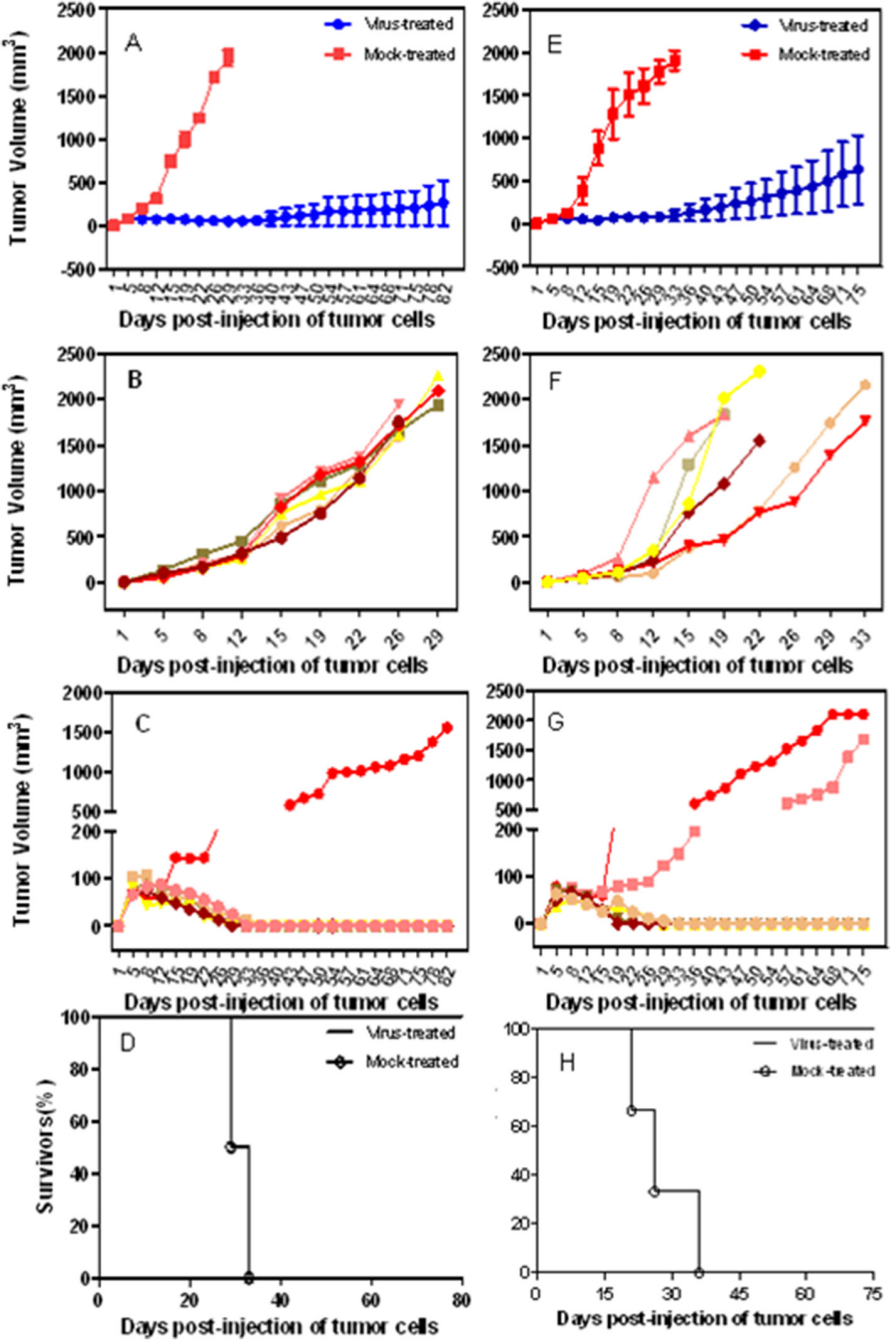
**Treatment of s.c. SMMC-7721 and BEL-7404 tumors with G47Δ.** The tumor size was measured by Vernier calipers, and the tumor volume was calculated (V = a × b^2^/2). The animals were sacrificed if they seemed moribund or the maximal diameter of their tumors exceeded 18 mm, and that day was recorded as the date of death for the survival analysis. G47Δ significantly delayed tumor progression compared with mock treatment. The tumors of 5 SMMC-7721 and 4 BEL-7404 mice in the treated groups regressed completely. **A**, The mean tumor volumes in the virus-treated and mock-treated SMMC-7721 groups at different times. **B** and **C**, The growth of individual tumors in the mock and virus treated SMMC-7721 groups. **D**, The median survival time for the mice with s.c. SMMC-7721 tumors was increased from 27.5 days for the mock-treated animals to over 82 days for the G47Δ-treated animals. **E**, The mean tumor volumes in the virus-treated and mock-treated BEL-7404 groups at different times. **F** and **G**. The growth of individual tumors in the mock and virus treated BEL-7404 groups. **H**, The median survival time for the mice with s.c. BEL-7404 tumors was increased from 21 days for the mock-treated animals to over 75 days for the G47Δ-treated animals.

Similar to the *in vitro* results, inoculation with G47Δ (2 × 10^7^ pfu, four times) elicited a prominent antitumor effect and significantly inhibited tumor growth. Following G47Δ treatment, complete regression was observed in 5 of 6 mice bearing s.c. SMMC-7721 tumors and in 4 of 6 mice bearing BEL-7404 tumors (Figure 4C, G). The animals were sacrificed on day 82 (for SMMC-7721) or day 75 (for BEL-7404), by which time the tumors in the specific group had grown to approximately 18 mm in maximal diameter. The G47Δ treatment significantly extended the survival of the mice bearing the s.c. SMMC-7721 and BEL-7404 tumors, with median survival times of 82 and 75 days, respectively, compared to median survival times of 27.5 and 21 days, respectively, in the mock treated groups (P < 0.05, log-rank test) (Figure 4D, H).

## Discussion

We evaluated the cytopathic effects of third-generation oncolytic HSV G47Δ on human HCC and immortalized hepatic cells. Additionally, we demonstrated the therapeutic value of HSV G47Δ in the treatment of HCC *in vivo*.

G47Δ has three deletions/mutations, γ34.5, UL39 and α47, that restrict replication to tumor cells. γ34.5 is required for HSV to replicate in the brain or cause encephalitis [31–33]. The protein product of γ34.5 precludes the halting of host protein synthesis in infected cells by dephosphorylating eIF2α [34]. γ34.5^−^ mutants lose neurovirulence, and their replication is attenuated in normal cells. However, the replication of γ34.5^−^ mutants is unaffected in dividing tumor cells. The virus is further mutated by a lacZ gene insertion into the gene encoding ICP6, which is required for efficient viral growth in non-dividing cells but not in many dividing cells in tissue cultures [35]. ICP6 is the large subunit of the viral ribonucleotide reductase, which is a key enzyme required synthesizing deoxyribonucleic acid in nondividing cells [35,36]. Without this enzyme, DNA replication is blocked in normal cells. Mammalian ribonucleotide reductase is elevated in tumor cells relative to normal cells; thus, the insertional inactivation of the gene encoding ICP6 guarantees the preferential replication of G47Δ in tumor cells [36]. Additionally, the mutation in the ICP6 gene makes the virus more sensitive to acyclovir and ganciclovir [37], which can augment its safety for clinical use. In addition to its direct cytotoxic effects, G47Δ is also capable of inducing antitumor immune responses [38–40]. The α47 gene binds to the transporter associated with antigen presentation (TAP) and blocks peptide loading of MHC class I molecules [41]. There is a α47 deletion in G47Δ [31]. This deletion increases MHC class I presentation, stimulates lymphocytes, and decreases NK cytolysis of infected cells, which can broaden the activation of an antitumor immune response [30]. This ability is an important feature for treating metastatic tumors, particularly in patients with severely limited treatment options. The α47 mutation also places the late US11 gene under the control of the immediate-early α47 promoter. This shift in control blocks the halting of host protein synthesis and results in amplified growth of γ34.5^−^ mutants and a boost in the cytotoxicity in tumor cells [34]. These multiple mutations make pathogenic reversion due to recombination nearly impossible and guarantee an important inherent safety mechanism for G47Δ treatment. The combination of safety and efficacy led us to explore G47Δ as a therapeutic agent for malignant tumors. Recently, G47∆ has entered phase 1 clinical trial for progressive glioblastoma.

Our results demonstrate that G47Δ can effectively kill different human HCC cell lines. Cytotoxic effects were observed in the HepG2, Hep3B, SMMC-7721, BEL-7404 and BEL-7405 cell lines. The efficiency of G47Δ in killing malignant HCC cells and inhibiting the growth of xenograft tumors, offers a promising therapeutic strategy in treating human hepatic tumors. As typical surgical resection may be not always possible or would induce serious complications, this new therapeutic treatment strategy for hepatic cancer should be of benefit to patients with hepatic cancer, especially those with late-stage cancer that have lost the opportunity of surgery. We also demonstrated the cytopathic effects of G47Δ on the HL-7702 human hepatic immortalized cell line at a very low MOI. Interestingly, immortalization of Schwann cells enhances their permissiveness to oncolytic HSV G207 [42]. Bypass of senescence and immortalization are considered to be early steps in tumor development, and thus G47Δ may be active at an early stage of tumorigenesis. So far, G47Δ has not posed a safety concern; thus, it could be used not only as a therapeutic strategy but also a preventive agent in pre-malignant diseases, such as hepatitis and cirrhosis.

To investigate the *in vivo* HCC antitumor effects of G47Δ, we established s.c. xenograft models of HCC using 2 different cell lines: SMMC-7721 and BEL-7404. G47Δ was administered to the Balb/c nude mice twice a week for 2 weeks without any observable toxicity. A higher survival rate and significant reduction in tumor growth relative to the mock-treated groups were observed after G47Δ therapy. Many of s.c. tumors from both the SMMC-7721 and BEL-7404 groups of G47Δ-treated Balb/c nude mice gradually regressed, suggesting that G47Δ has the potential to effectively inhibit different types of HCC tumors in clinical applications.

In conclusion, the third-generation oncolytic HSV G47Δ was effective as a tumoricidal agent in both HCC and immortalized hepatic cells. This feature suggests its use as both a therapeutic and preventive agent for human HCC. Intratumoral injection of G47Δ induced an obvious therapeutic effect on HCC, which may lead to future clinical applications in cancer therapy.
